# Lessons learned from the first 50 COVID-19 critical care transfer missions conducted by a civilian UK Helicopter Emergency Medical Service team

**DOI:** 10.1186/s13049-022-00994-7

**Published:** 2022-01-15

**Authors:** J. Jeyanathan, D. Bootland, A. Al-Rais, J. Leung, J. Wijesuriya, L. Banks, T. Breen, R. DeCoverly, L. Curtis, A. McHenry, D. Wright, J. E. Griggs, R. M. Lyon

**Affiliations:** 1 Air Ambulance Kent Surrey Sussex, Redhill Aerodrome, Redhill, Surrey RH1 5YP UK; 2 University of Surrey, Guildford, UK

**Keywords:** COVID-19, Critical care, Transfer medicine, Helicopter Emergency Medical Services, Intensive care

## Abstract

**Background:**

The COVID-19 pandemic has placed exceptional demand on Intensive Care Units, necessitating the critical care transfer of patients on a regional and national scale. Performing these transfers required specialist expertise and involved moving patients over significant distances. Air Ambulance Kent Surrey Sussex created a designated critical care transfer team and was one of the first civilian air ambulances in the United Kingdom to move ventilated COVID-19 patients by air. We describe the practical set up of such a service and the key lessons learned from the first 50 transfers.

**Methods:**

Retrospective review of air critical care transfer service set up and case review of first 50 transfers.

**Results:**

We describe key elements of the critical care transfer service, including coordination and activation; case interrogation; workforce; training; equipment; aircraft modifications; human factors and clinical governance. A total of 50 missions are described between 18 December 2020 and 1 February 2021. 94% of the transfer missions were conducted by road. The mean age of these patients was 58 years (29–83). 30 (60%) were male and 20 (40%) were female. The mean total mission cycle (time of referral until the time team declared free at receiving hospital) was 264 min (range 149–440 min). The mean time spent at the referring hospital prior to leaving for the receiving unit was 72 min (31–158). The mean transfer transit time between referring and receiving units was 72 min (9–182).

**Conclusion:**

Critically ill COVID-19 patients have highly complex medical needs during transport. Critical care transfer of COVID-19-positive patients by civilian HEMS services, including air transfer, can be achieved safely with specific planning, protocols and precautions. Regional planning of COVID-19 critical care transfers is required to optimise the time available of critical care transfer teams.

## Background

The coronavirus (SARS-CoV-2) pandemic (COVID-19) has challenged health systems across the globe [1]. In particular, a major demand has been placed on critical care facilities. A significant proportion of COVID-19 patients required treatment with critical care interventions, including ventilatory support [2]. This unprecedented demand led to Intensive Care Unit (ICU) resources being put under significant strain on both regional and national levels. At a local level, ICU bed pressures necessitated the rapid creation of acute surge capacity. Despite these expanded footprints, the critical care capacity in many hospitals remained under significant pressure. In order to preserve standards of critical care and mitigate these demands, it became necessary for hospitals experiencing acute demand to request critical care transfers to other ICUs, utilising system resources across the region and then beyond. During the height of the pandemic in early 2021, there were several requests on a daily basis within our region requesting critical care transfers of COVID-19 patients. These demands could not be met by the existing hospital workforce. The unprecedented level of demand led to resource strain at both regional and national levels and mandated the creation of de novo critical care transport teams in order to maintain equitable access to intensive care. The number of necessitated transfers also meant that many of these were undertaken over large distances to other regions [3].

The demand for critical care transfers during the height of the pandemic was unprecedented [2]. Emergency Medical Services (EMS) with experience and capability to undertake critical care transfers were asked, at very short notice, to increase their capacity and adapt to being able to transfer critically unwell COVID-19 positive patients. The highly infectious nature of COVID-19, particularly in relation to performing Aerosol Generating Procedures (AGPs), required specific protective measures to be taken to safely transfer COVID-19 patients, without putting EMS or associated personnel, such as pilots, at risk [4]. In the UK, pre-hospital critical care teams such as Helicopter Emergency Medical Services (HEMS) have adapted, overcome, and continued to deliver high acuity trauma and medical care to patients at their time of need. In addition, several HEMS services rapidly adapted to provide a critical care transfer capability. Indeed, the combination of highly experienced senior clinicians working within a mature governance framework alongside an established transport platform, lent itself well to HEMS services adapting to undertake work of this nature.

Critical care transfer medicine has several essential areas which require careful consideration [5]. These considerations were especially highlighted in the context of ICU-level COVID-19 patients, due to their need for complex multi-organ support, particularly advanced ventilatory support, and their physiological fragility. The challenge of these transfer cases was often exacerbated by short notice, urgent referrals for transfers over significant distances and the need for escorting clinicians to wear level 3 /ICU Personal Protective Equipment (PPE) throughout. This paper provides a descriptive overview of how our UK HEMS service, in collaboration with our local National Health Service (NHS) ambulance provider (South East Coast Ambulance Service NHS Foundation Trust—SECAmb), rapidly evolved to provide an aeromedical transfer capability for COVID-19 patients. We present a pragmatic review of the first 50 COVID-19 transfers undertaken by Air Ambulance Kent Surrey Sussex and highlight key lessons learned that would be useful to other EMS services tasked with setting up such a service.

## Methods

Air Ambulance Kent Surrey Sussex (AAKSS) delivers care to a mixed urban and rural area, covering 4.5 million people across the south east of England. The HEMS team comprises of an experienced physician and paramedic, capable of delivering enhanced care, including pre-hospital emergency anaesthesia, blood product administration, procedural sedation and emergency surgery. These interventions cannot be routinely performed by land ambulance crews. The HEMS service operates from two separate bases, responds 24/7 and can respond in either a helicopter or response car, depending on geography and weather limitations. Patients are transported to hospital either by helicopter or land ambulance.

In December 2020, a so-called “Kent” variant (subsequently known as B.1.1.7.) of COVID-19 which appeared more contagious than other variants, rapidly spread through the south east of England, the region which AAKSS serves. The number of critically unwell patients rapidly challenged the intensive care unit (ICU) capacities within many of the hospitals of Kent, Surrey and Sussex. As part of a national strategy, overseen by the NHS, to maintain equitable access to critical care, coordination and provision of a robust critical care transfer capability became a necessity [3]. With a notice period of just a few weeks, AAKSS developed a Critical Care Transfer Service to dovetail with its primary pre-hospital emergency medicine (PHEM) duties. To build in layers of safety, a number of standardised processes were rapidly implemented.

At the time of implementation, ICUs in the south east of England were under unprecedented pressure. There would often be several patients on particular hospital sites requiring admission to ICU when the local unit was already at capacity. To manage system capacity, patients were transferred between ICUs, with the most stable patients being selected for transfer. These would often involve non-COVID patients.

We review the steps required to set up an aeromedical transfer service capable of safely and robustly moving Level 3 COVID-19 positive patients; the training, operational and medical elements needed to deliver such a service safely and effectively and we present key lessons learned from the first 50 COVID-19 transfers. The key elements were based on internal expert opinion and we sought to present a pragmatic, descriptive approach to inform other pre-hospital services involved in the transport of COVID-19 patients.

## Results

The key elements that needed to be established for a dedicated aeromedical transfer service to launch are described below. These elements were considered by all authors as the most important when having to rapidly adapt from primary HEMS work to secondary COVID transfers.

### Coordination and activation of a specifically tailored and rehearsed level 3 COVID-19 transfer process

**Fig. 1:**
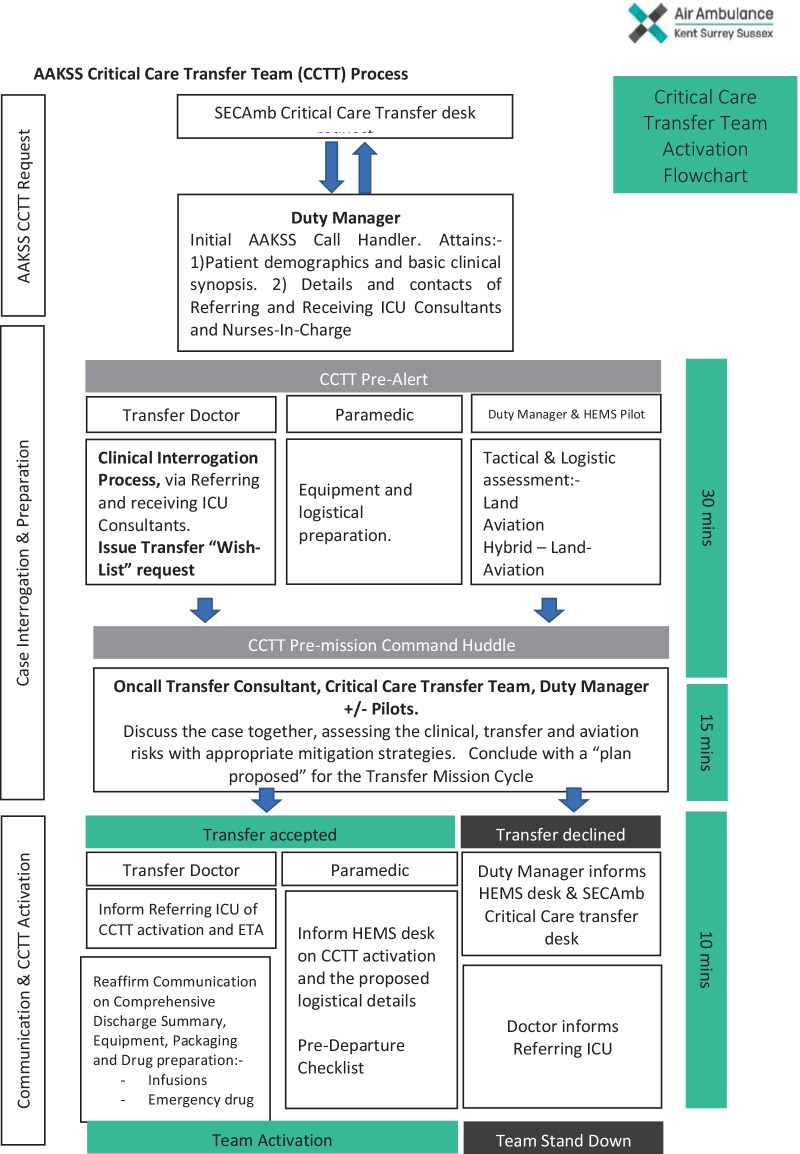
AAKSS critical care transfer process

Transfer requests were identified via a central process to SECAmb following a daily regional meeting and subsequently passed to the AAKSS Duty Clinical Manager. This would commence a chain of defined concurrent activity in order to plan the conduct of the tasking. Each individual transfer request was overseen by the Duty Clinical Manager and an on-call AAKSS HEMS Transfer Consultant (with experience in both pre-hospital emergency medicine and current ICU COVID-19 care). The Duty Transfer crew consisted of a Transfer Doctor (who was an AAKSS HEMS doctor from an ICU-Anaesthesia specialty) and an AAKSS Transfer Paramedic. The temporal nature of identifying and tasking a transfer following the receipt of requests after regional meetings meant that transfers typically occurred in the afternoon and evening. The process overview is shown in Fig. 1.

### Case “Interrogation” process

**Fig. 2:**
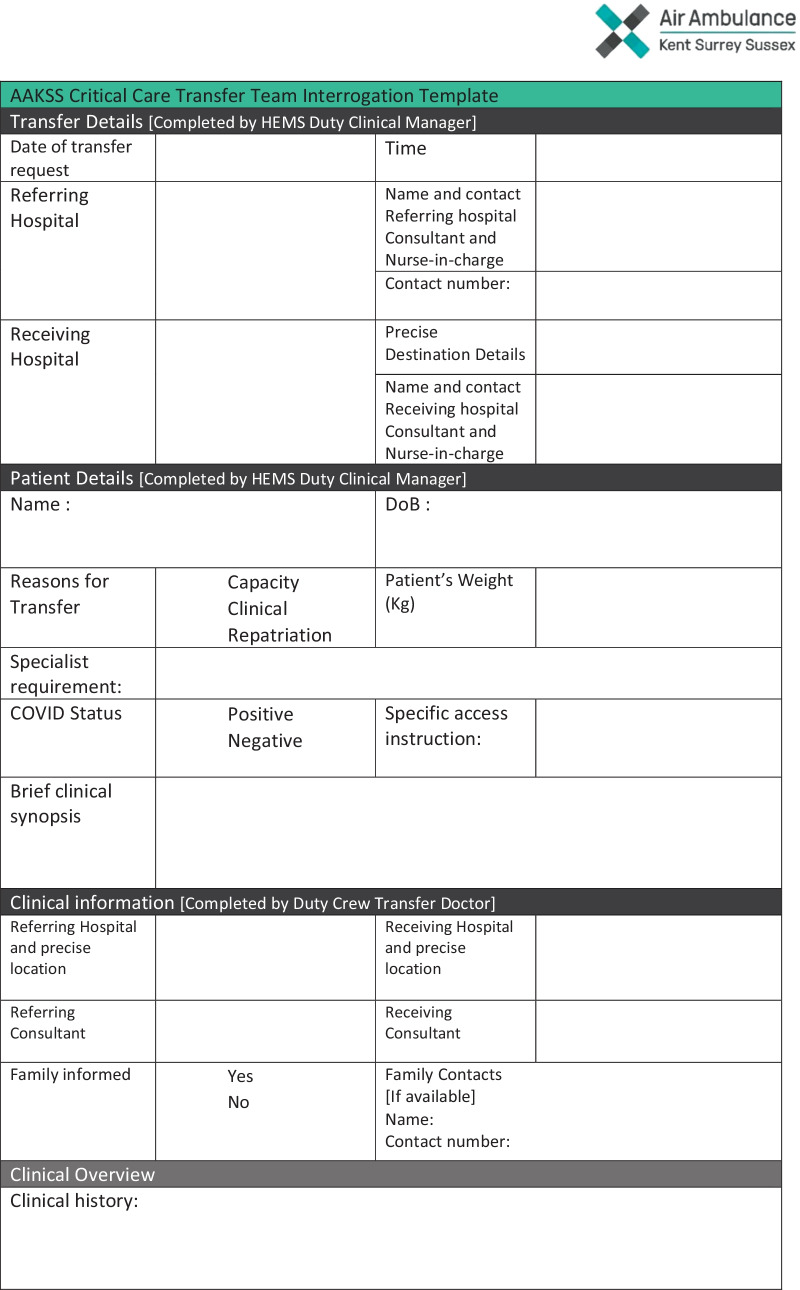
AAKSS critical care transfer planning sheet

Requests for COVID-19 transfers were coordinated at regional level by the NHS England Improvement critical care coordination cell and SECAmb. Each request was considered on an individual basis by AAKSS. A patient selection proforma utilised a specifically tailored case interrogation template, as shown in Fig. 2. The complexity, instability and physiological fragility of COVID-19 patients meant that rigorous clinical interrogation, with case-by-case consideration of the challenges posed by moving these patients was required on each occasion.

Prior to deploying on a transfer tasking, a “command huddle” was conducted. At the command huddle the transfer team, duty transfer consultant and duty clinical manager would appraise the clinical and logistic aspects of the case, identify potential risks and pitfalls, discuss mitigation strategies, and decide the most appropriate course of action. Particularly complex or high risk transfers were escalated to the Medical Director for further review and final decision making.

### Workforce

The AAKSS crew was a doctor and paramedic. The doctors were all experienced, long-standing AAKSS PHEM doctors and were additionally Consultants in Anaesthesia and Intensive Care Medicine and had recent and regular ongoing exposure to patients who were critically unwell with COVID-19. The AAKSS paramedics had undertaken concurrent training in specific elements of critical care and COVID-19 [6]. This specific crew configuration allowed for a familiarity in caring for the critically unwell patient in the out-of-hospital environment. Familiarity between members of the workforce was a particularly important factor in overcoming the additional and significant challenges posed by operating in full Level 3 PPE. Personal protective equipment need to be in-line with standard hospital practice including eye protection, FFP3 masks and surgical gowns. Crews were also given the option of wearing Positive Airway Pressure Respirator hoods.

### Training

A competency-based critical care transfer training module was developed and instituted to ensure specific training and currency in critical care practice and the management of patients with multi-organ dysfunction. Training built upon the pre-existing PHEM practices and expertise and, as the service developed, was further enhanced, and standardised to a formal training pathway for Critical Care Transfer Medicine. All transfer team members undertook a HEMS Transfer Training day, alongside a half day Critical Care COVID Transfer Medicine Package, with a specific focus on the physiology, pharmacology and practical techniques required to manage complex and critically ill patients. Individual crew members were then required to complete a curriculum of core clinical topics, equipment competencies and logistic considerations. It took varying amounts of time for crew members to develop competence and confidence in critical care transfer and while there was no set time to complete the training log, 2–4 weeks was suggested. During this period the crew member also undertook at least four transfer shifts under the supervision of a Transfer Consultant. Training culminated in a full day sign-off assessment, including a clinical viva, equipment test and clinical long case discussion.

### Equipment

A specific, dedicated set of transfer equipment and bags were assembled. The content was based on the need to maintain the highest standards of intensive care throughout the duration of the transfer. Ventilation was provided with a Dräger Oxylog 3000 ventilator in line with our primary HEMS work and monitoring maintained using the Tempus Pro Monitor (Phillips RDT). This allowed for the added advantage of recording physiological data directly into the electronic clinical record. Infused medicines were delivered via Braun perfusor syringe drivers. Using identical equipment to that used in primary HEMS work was an important consideration in order to enhance the safety of this type of work and minimise the cognitive load that comes with managing patients of this complexity. The transfer kit was physically entirely separate to the HEMS kit and could be deployed onto a land ambulance or helicopter.

### Transfer platform and infrastructure

All critical care transfers were considered for transfer via land, air or a land-air hybrid. Given the complicated geography in our region, with a mix of urban areas within rural and coastal settings, the potential opportunity for air or hybrid transfer mission cycles allowed an enhancement in care by decreasing the period a sick COVID-19 patient was out of a hospital ICU environment. This also accelerated the regeneration of the critical care transfer crew. Several transfers, including the long distance mission cycles, whilst considered for air transfers, often resulted in either pure land or hybrid transfers. Overall, 94% of the transfer missions were conducted by land. This was due to the time of year being winter (December-February), with both light and weather restrictions, which made long distance critical care transfers by air using visual flight rules challenging to undertake.

### Care of the COVID-19 patient during transfer

Meticulous handling of the COVID-19 patient was required prior to, during, and after transfer. Respiratory failure was the overwhelming organ failure, requiring multi-faceted management strategies, particularly for refractory hypoxia. Stabilising the patient on the transport ventilator was a particular challenge for some patients and was typically attempted early in the transfer process. In practice, our transfer team most commonly encountered pressure-controlled ventilation. Our team mirror the pressure setting as the initial step of ventilator transition. We then closely observe the changes of the patient’s minute volume. If minute volume reduced, our team will implement an incremental increase to inspiratory pressure until the desired minute volume is achieved. We allowed permissive hypercapnia. We obtained an arterial blood gas sample 15 min after the transition to the transport ventilator (Oxylog 3000). To avoid patient-ventilator asynchrony during the mission, deep sedation and paralysis were used for the entire transfer journey.

**Fig. 3:**
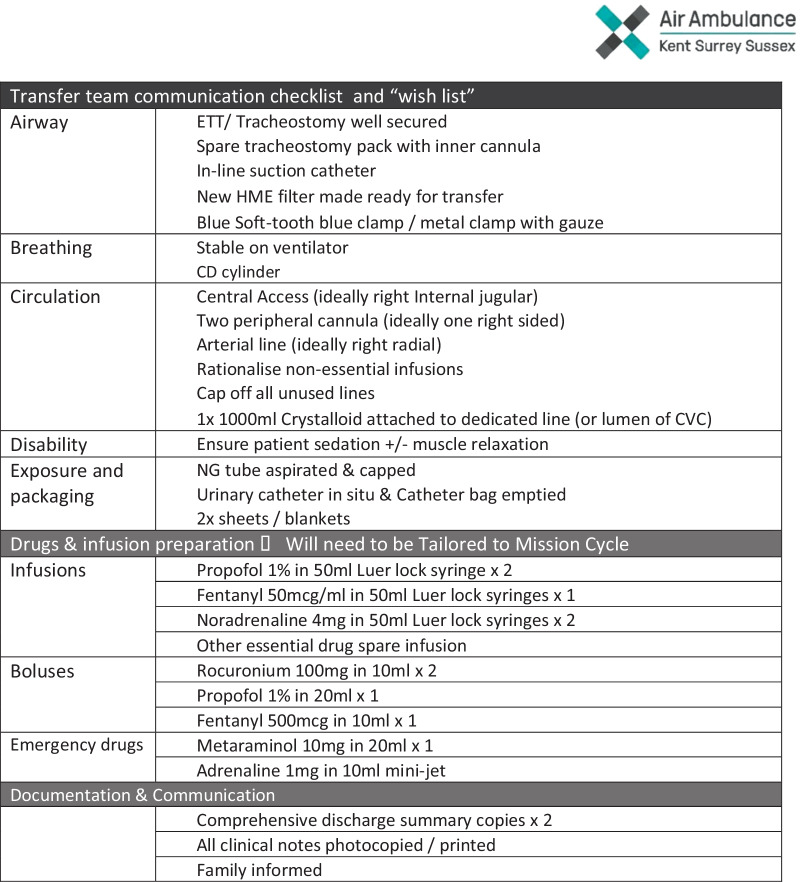
Transfer team communication checklist

The complex implications of COVID-19 on the vascular structure and haematological dynamics, often with a pro-thrombotic propensity, gave these patients a uniquely precarious physiological fragility. The interrogation process between the referring ICU consultant and transfer team was important, but a further dynamic assessment of the patient was essential on transfer team arrival at the referring ICU. Gentle bridging on to transfer specific infusion pumps, ventilator, monitoring and bed was essential followed by careful handling of the patient’s complex pathophysiology. Pre-arrival requests were structured, as shown in Fig. 3.

### Interpersonal relationships, human factors and communication

The management of a COVID-19 patient is made harder by the need to work carefully in full PPE. Clear communication was therefore imperative. As a Critical Care Transfer Team, it was important to forge relationships with referring critical care teams, clearly communicate with the receiving ICU and work cohesively alongside a number of new groups of health professionals and team members. The primary PHEM training and practices, particularly in crew resource management (CRM) and communication skills, proved a core strength and foundation for the critical care transfer capability response.

### Aircraft modification

To protect the pilots, a sealed barrier curtain was installed between the cockpit and cabin section of the AW169 helicopter. This achieved a hermetic seal with different air supplies to the pilot and patient cabin sections. Pilots flew with standard surgical masks, following testing and approval of radio communications whilst wearing them. The size and specification of the AW169 cabin allowed for excellent access to the patients throughout flight, and the ability to maintain monitoring and titrate infusions presented no problems. A closed suction system allowed for in-flight suction of the trachea if required.

### Clinical Governance

A specific Clinical Governance framework was established that mirrored that of AAKSS primary missions but stood as an independent framework. Whilst this specific process was created de novo to address a specific challenge in the critical care transfer of level 3 COVID-19 patients (Fig. 1), it was embedded in a mature system of standard operating procedures, governance and logistics. A dedicated Transfer Consultant was on-call for remote support and all cases underwent detailed case review.

## Analysis of the 50 critical care transfers during the UK COVID-19 s wave

Between 18 December 2020 and 1 February 2021, AAKSS in collaboration with SECAmb performed 50 adult critical care transfers in support of the UK COVID-19 response.

All 50 of these critical care transfers were undertaken to urgently help with ICU capacity across the region. These ICUs were all managing patient numbers significantly beyond their normal footprint. As a result, through a nationally coordinated initiative, other ICUs with capacity were identified to provide mutual aid, often in areas a significant distance away.

Of the 50 critical care transfers, 45 (90%) were critically unwell patients receiving Level 3 multi-organ support. 5 (10%) patients were receiving Level 2 care and all of these were for non-COVID-19 disease processes. All missions had data entered in real time into the AAKSS patient record/mission data system (HEMSbase, Medic One Systems Ltd). A specific section had already been created to record secondary transfer missions. All data were then analysed retrospectively.

The mean age of these patients was 58 years (range 29–83). 30 (60%) were male and 20 (40%) were female.

The AAKSS aircraft was used for 3 (6%) transfers and 47 (94%) were moved by road. To our knowledge, this represented the first civilian air transfers of COVID-19 positive patients in the UK.

All of these patients were invasively ventilated with mandatory or pressure support ventilation. 45 (90%) of these patients had an endotracheal tube in situ and 5 (10%) had a tracheostomy sited to facilitate weaning from mechanical ventilation.

The mean FiO_2_ at referral was 0.45 (0.21–0.8). 17 (34%) patients were established on vasopressor support at the point of referral, versus 33 (66%) on no cardiovascular support. All 17 patients on vasopressor support were receiving noradrenaline, with 1 patient also receiving dobutamine.

The mean total mission cycle (time of referral until the time team declared free at receiving hospital) was 264 min (range 149–440 min). The mean time spent at the referring hospital prior to leaving for the receiving unit was 72 min (31–158). The mean transfer transit time between referring and receiving units was 72 min (9–182).

During this period, no significant adverse events occurred and there were no instances of transfer team members or pilots contracting COVID-19 as a result of a transfer mission.

## Discussion

AAKSS successfully implemented a fully functional critical care transfer service capable of moving critically ill COVID-19 patients by air. The majority of these patients were in multi-organ failure due to COVID-19 infection. The adherence to a standardised pathway with an interrogation process allowed for an efficient service, which focussed on patient safety. The investment in the workforce and subsequent crew configuration was labour intensive, but ensured a robust and consistent service. The training elements and governance were imperative in ensuring responsive practices, especially as our clinical approach to COVID-19 evolved. Having a dedicated AAKSS Transfer Consultant and the ability to activate “Command Huddles” throughout a critical care mission were both useful for patient care but also for supporting crews and fostering interpersonal relationships across healthcare providers during a very challenging time. The authors of this paper would recommend using these interventions, which we believe enhanced mission and organisational safety.

The need for local, regional and national coordination of critical care assets and transfer requirements is imperative for future pandemic initiatives. To optimise the available time of the critical care transfer teams, planning should ideally occur on an ongoing basis, with patients identified for transfer the preceding night. This allows transfer teams to maximise their impact. This is particularly important for aeromedical teams who may be better operated in daylight conditions. The number of COVID-19 transfers conducted by air was limited, largely by environmental factors. As familiarity and efficiency of the transfer systems evolves, we anticipate increased air transfers.

To our knowledge, AAKSS was the first civilian air ambulance service to move COVID-19 patients by air in the UK. This was achieved through early engagement with the required authorities to gain approval for the safety procedures put in place to protect pilots from the risk of infection. The use of an aeromedical transport platform has the potential to confer a significant advantage for patients moved over large distances. Any concerns regarding the potential physiological insult posed by altitude are negligible by helicopter transport in our region, with flights undertaken at around 1000 ft above sea level.

Our teams spent a significant amount of time on arrival at the referring hospital when compared to our scene times for primary HEMS work. The time was largely due to the physiological complexity of COVID-19 patients, including, for example, the careful transfer of the patient from an ICU to a transport ventilator and the associated interventions required to ensure a safe and stable critical care transfer. In this particular example, although the assessment of stability and suitability to be moved on a transport ventilator could be streamlined by having the referring hospital undertake ventilator exchange prior to transfer team arrival.

Other pre-hospital services have published their experience of transferring critical COVID-19 patients [1, 7]. Several providers described the effective use of patient isolation units (PIU) [1]. AAKSS was concerned about the limitations of being able to treat critically unwell patients whilst in a PIU and therefore focussed on securing the entire rear of the helicopter to prevent infection. Similar to other published research, only a minority of transfers were completed by air, highlighting the technical challenges of air transport of COVID-19 patients. However, for long distance transfer of COVID-19 patients, air is likely to be the faster and more effective transport platform [8].

We recognise that this is a relatively small descriptive study over a short time period. We acknowledge that our experience will not necessarily be applicable to all services, particularly outside the UK. However, we have demonstrated a method for a HEMS service to rapidly, effectively and safely stand up a critical care transfer service capable of moving level 3 COVID-19 patients by both land, air or a hybrid model. We have shown this is possible in the civilian setting and the policies and protocols presented in this paper will likely be useful to other services.

Further research is warranted, particularly with regards PPE and how best to prevent cross-infection during transfer of COVID-19 patients [9, 10]. As further waves of COVID-19 patients stretch emergency medical services globally, sharing of experience will be invaluable.

## Conclusions

The COVID-19 pandemic has placed unprecedented pressures on critical care resources, necessitating the rapid establishment of adult critical care transfer services to decompress overwhelmed hospitals, to support clinicians and minimise preventable loss of life due to resource depletion. Critically ill COVID-19 patients have highly complex medical needs during transport. Critical care transfer of COVID-19 positive patients by civilian HEMS services, including air-transfer, can be achieved safely with specific planning, protocols and precautions. Regional planning of COVID-19 critical care transfers is required to optimise the time available of critical care transfer teams.

## Data Availability

All data is presented.

## Abbreviations

AAKSS: Air Ambulance Kent Surrey Sussex
AGP: Aerosol generating procedure
EMS: Emergency Medical Service
HEMS: Helicopter Emergency Medical Service
ICU: Intensive Care Unit
PHEM: Pre-hospital emergency medicine
PIU: Patient isolation unit
PPE: Personal protective equipment
SECAmb: South East Coast Ambulance Service Trust
